# Consequences of habitat fragmentation on the reproductive success of two *Tillandsia* species with contrasting life history strategies

**DOI:** 10.1093/aobpla/ply038

**Published:** 2018-06-19

**Authors:** Roberto Sáyago, Mauricio Quesada, Ramiro Aguilar, Lorena Ashworth, Martha Lopezaraiza-Mikel, Silvana Martén-Rodríguez

**Affiliations:** 1 Facultad de Desarrollo Sustentable, Campus Costa Grande, Universidad Autónoma de Guerrero, Carretera Nacional Acapulco Zihuatanejo Km, Colonia Las Tunas, Tecpan de Galeana, Guerrero, México; 2 Laboratorio Nacional de Análisis y Síntesis Ecológica (LANASE), Escuela Nacional de Estudios Superiores (ENES), Universidad Nacional Autónoma de México, Antigua Carretera a Pátzcuaro, Morelia, Michoacán, C.P., México; 3 Instituto de Investigaciones en Ecosistemas y Sustentabilidad, Universidad Nacional Autónoma de México, Antigua Carretera a Pátzcuaro, Morelia, Michoacán, C.P., México; 4 Instituto Multidisciplinario de Biología Vegetal (CONICET), Universidad Nacional de Córdoba, C.P. X5000JJC, Argentina

**Keywords:** Bromeliaceae, fragmentation, hummingbird pollination, monocarpy, polycarpy, reproductive success

## Abstract

Fragmentation of natural habitats generally has negative effects on the reproductive success of many plant species; however, little is known about epiphytic plants. We assessed the impact of forest fragmentation on plant–pollinator interactions and female reproductive success in two epiphytic *Tillandsia* species with contrasting life history strategies (polycarpic and monocarpic) in Chamela, Jalisco, Mexico, over three consecutive years. Hummingbirds were the major pollinators of both species and pollinator visitation rates were similar between habitat conditions. In contrast, the composition and frequency of floral visitors significantly varied between habitat conditions in polycarpic and self-incompatible *T. intermedia* but not in monocarpic self-compatible *T. makoyana*. There were no differences between continuous and fragmented habitats in fruit set in either species, but *T. makoyana* had a lower seed set in fragmented than in continuous forests. In contrast, *T. intermedia* had similar seed set in both forest conditions. These results indicate that pollinators were effective under both fragmented and continuous habitats, possibly because the major pollinators are hummingbird species capable of moving across open spaces and human-modified habitats. However, the lower seed set of *T. makoyana* under fragmented conditions suggests that the amount and quality of pollen deposited onto stigmas may differ between habitat conditions. Alternatively, changes in resource availability may also cause reductions in seed production in fragmented habitats. This study adds to the limited information on the effects of habitat fragmentation on the reproductive success of epiphytic plants, showing that even related congeneric species may exhibit different responses to human disturbance. Plant reproductive systems, along with changes in pollinator communities associated with habitat fragmentation, may have yet undocumented consequences on gene flow, levels of inbreeding and progeny quality of dry forest tillandsias.

## Teaser

This study assessed the impact of habitat fragmentation on plant–pollinator interactions and reproductive success of two epiphytic Mexican *Tillandsia* species over 3 years. Pollinator assemblages changed in species composition between fragmented and continuous habitats for *T. intermedia*, but hummingbirds—the most important pollinators of both *Tillandsia* species—were effective under both habitat conditions, reflecting their ability to move across human-modified landscapes. *Tillandsia makoyana* had a lower seed set under fragmented conditions, suggesting that differences in pollination quality between habitat conditions may have undocumented consequences on the quality progeny of these dry forest *Tillandsia* species.

## Introduction

Anthropogenic loss and fragmentation of natural habitats have profound consequences on the structure of biological communities and populations (Fahrig 2004). In plants, habitat fragmentation may impact the genetic and demographic structure of populations resulting in local or species-level extinctions, but more commonly, fragmentation has effects on plant growth and reproduction through changes in the biotic and abiotic environments (Honnay *et al.* 2005; Aguilar *et al.* 2008; Quesada *et al.* 2011). Thus, long-term persistence of plant populations in fragmented habitats ultimately depends on the effect habitat fragmentation has on abiotic factors like soil and microclimate, and on biotic processes, such as herbivory, pollination and seed dispersal (Bawa *et al.* 2003; Fuchs *et al.* 2003; Mix *et al.* 2006). A large number of studies have tested the impact of habitat fragmentation on pollination and plant reproductive success, many of them in tropical angiosperms; however, vascular epiphytes have received considerably less attention (Aguilar *et al.* 2006; Parra-Tabla *et al.* 2011).

Epiphytes are non-parasitic plants that grow on other plants (phorophytes) and they represent almost 10 % of vascular plant species (Benzing 1990; Zotz 2016). Vascular epiphytes are important elements of tropical forests due to their high taxonomic and functional diversity, and their role in supplying nutrients, water and shelter for other organisms (Zotz 2016). Moreover, epiphytes represent an important proportion of the total biomass in tropical forests, playing an essential role in forest nutrient fluxes and water retention (Hofstede *et al.* 1993; Zotz and Bader 2009). Epiphytes are considered particularly sensitive to habitat disturbance because they have low growth rates, delayed sexual maturity, limited seed dispersal and recruitment, and no seed bank (Turner *et al.* 1994; Martin *et al.* 2004; Cascante-Marín *et al.* 2009). In terms of habitat fragmentation, most studies on epiphytic plants show that the abundance, diversity, growth, dispersal and genetic parameters of epiphytes are negatively affected by fragmentation (González-Astorga *et al.* 2004; Flores-Palacios and García-Franco 2004; Werner and Gradstein 2008, 2009; Aguirre *et al.* 2010). This has been related to the dependence of epiphytes on their host trees, which makes epiphytes vulnerable to changes in the availability and traits of their phorophytes (Magrach *et al.* 2012; Sáyago *et al.* 2013). Surprisingly, the impact of habitat fragmentation on the pollination and reproductive success of vascular epiphytes is poorly known, except for a number of orchid species (e.g. Parra-Tabla *et al.* 2000,^2011^; Murren 2002), and few species in the Cactaceae and the Bromeliaceae families (Aizen and Feinsinger 1994).

One aspect that deserves attention in studies of habitat fragmentation is related to the response of plants to habitat changes with respect to variation in plant life history strategies. In general terms, polycarpic or iteroparous species have more than one reproductive event during their lifetimes, whereas monocarpic or semelparous species have a single reproductive event, after which they die (Amasino 2009). Associated with each life history strategy is the number of offspring produced by reproductive event, which is generally higher in monocarpic than in polycarpic species (Cole 1954). Furthermore, monocarpic species often have breeding systems that allow them to reproduce in the absence of pollinators, such as autonomous self-pollination (Cole 1954; Brys *et al.* 2011). Monocarpism is generally associated to annual herbs, but it is also present across all plant life forms, including a reduced number of long-lived trees and epiphytic species (Benzing 2000; Poorter *et al.* 2005). While monocarpism is rare in epiphytes, it apparently evolved more than once in epiphytic bromeliads of the genus *Tillandsia* (Young and Augspurger 1991). Nonetheless, evidence is lacking for how long-lived monocarpic epiphytes respond to habitat fragmentation.

Bromeliads provide a relevant study system to assess the impact of habitat fragmentation on the reproduction of epiphytic plants as they are one of the most important components of vascular epiphyte communities in the Neotropics (Benzing 2000). In this study, we focused on two tropical dry forest *Tillandsia* species with contrasting life histories to determine the impact of habitat fragmentation on plant–pollinator interactions and the reproductive success of epiphytes with monocarpic and polycarpic life history strategies. To accomplish this, we compared pollinator assemblages, pollinator visitation rates, flower production, fruit set and seed set between fragmented and continuous habitats of a tropical dry forest across 3 years. We expected a greater reproductive display per individual in the monocarpic than in the polycarpic species associated with a greater pollinator visitation and therefore, little or no effect of habitat fragmentation on fruit and seed set. For the polycarpic species, we expected that lower pollinator visitation would lead to greater temporal variance in reproductive success and possibly a negative effect of fragmentation on reproduction.

## Methods

### Study site

**Figure 1.**
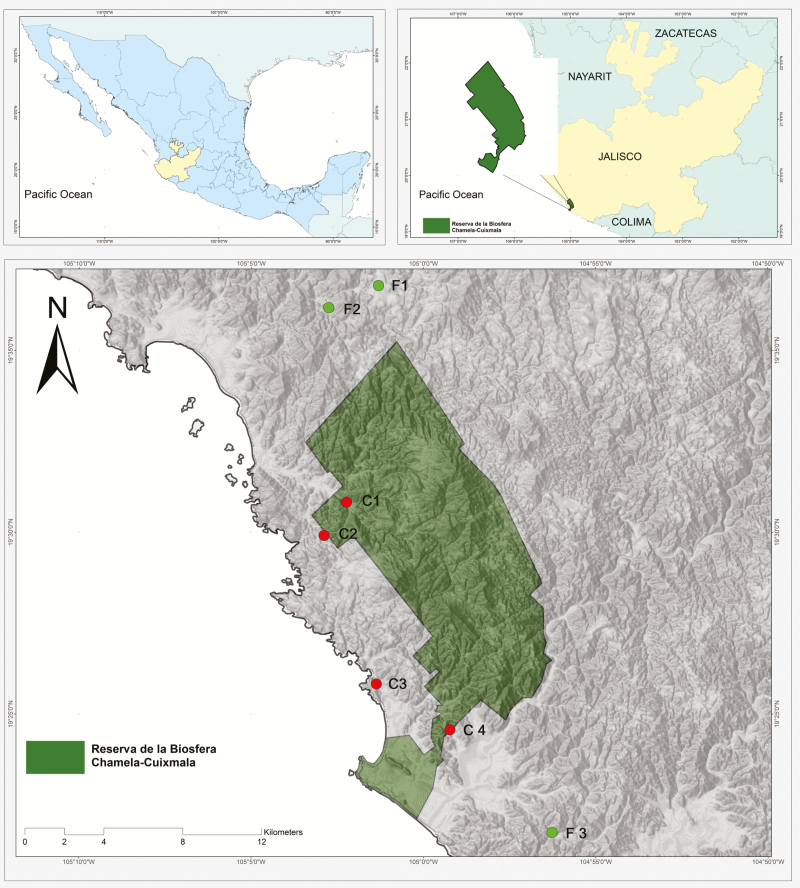
Map of the region of Chamela, Jalisco, Mexico, showing the continuous sites (C1–4) and fragmented sites (F1–3) where *Tillandsia intermedia* and *T. makoyana* were studied during 2008–10.

The study was conducted in the region of Chamela, state of Jalisco, Mexico, in the northernmost natural protected area of tropical dry forest in the Americas and surrounding areas (Fig. 1). Climate in this region is strongly seasonal with a wet season extending between July and October and a long dry season the rest of the year. Mean annual rainfall is 748 mm and mean annual temperature is 24.9 °C (García-Oliva *et al.* 2002). This location is one of the most species rich of all Neotropical dry forests including a number of species that are endemic to the Pacific coast of Mexico (Lott and Atkinson 2006). The most species-rich family is Fabaceae, followed by Euphorbiaceae and Convolvulaceae; the Bromeliaceae with 22 species occupies the sixth place in species richness in Chamela (Gentry 1995). Along the Pacific coast of Mexico, tropical dry forest habitats have been exposed to high levels anthropogenic disturbance (Trejo and Dirzo 2000; Quesada and Stoner 2004). Cattle ranching and agriculture have fragmented most of the forest in the study region, except for the Chamela-Cuixmala Biosphere Reserve (13142 ha; Sanchez-Azofeifa *et al.* 2008). Fragmentation of the continuous forest in this region was promoted in the 1950s under a government programme that advocated colonization of the Pacific coast of Mexico (Quesada *et al.* 2014).

### Study species

The genus *Tillandsia* is the second most species-rich genus of the whole flora of Chamela. The study species: *Tillandsia intermedia* and *T. makoyana*, are both in the subgenus *Tillandsia*. These species were selected due to their contrasting reproductive strategies (monocarpic vs. polycarpic), shared pollinators (hummingbirds) and similar flowering seasons. Across the three study years, populations of both *Tillandsia* species flowered from April to early July each year, at all sites. Fruits develop and mature 1 year after flowering (Sáyago 2016).

**Figure 2.**
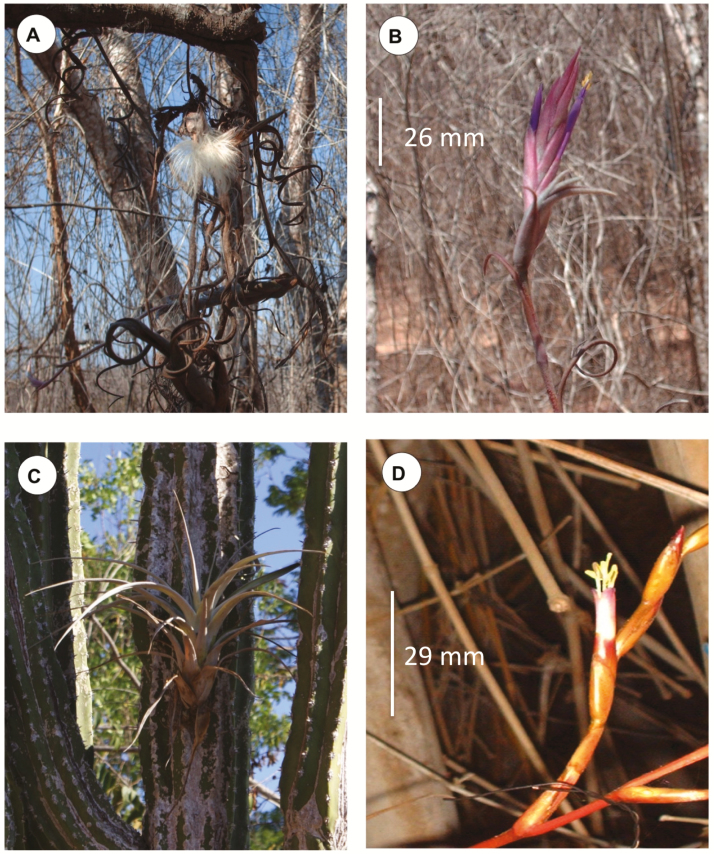
Plants and flowers of polycarpic *Tillandsia intermedia* and monocarpic *T. makoyana* from the region of Chamela, Jalisco, Mexico. Ramets of a single genet of *T. intermedia* dispersing seeds (A) and inflorescence showing one open flower (B); *T. makoyana* single ramet plant (C) and flower (D).

*Tillandsia intermedia* is endemic to Mexico, mostly distributed along the central Pacific coast of the country (https://www.gbif.org/species/2695234). It is a polycarpic, clonal herb, characterized by hollow pseudobulbs that are formed by overlapping leaf bases; leaf blades are involute, and contorted (Fig. 2A). Inflorescences are spikes with pink tubular, perfect flowers that produce on average seven flowers (Sáyago 2016; Fig. 2B); anthesis lasts 1 day and flowers produce on average 0.7 μL of nectar (Arizmendi and Ornelas 1990; syn. *T. paucifolia*). This species has the potential for secondary dispersal by asexual means, when ramets (clones) detach.

*Tillandsia makoyana* is distributed from Mexico to Costa Rica. It is a monocarpic tank bromeliad with leaves up to 70 cm long (Fig. 2C). Inflorescences are long compound spikes that produce on average 84 perfect flowers with light-purple tubular corollas (Fig. 2D); anthesis lasts 1 day and flowers produce, on average, 7 μL of nectar per day. *Tillandsia makoyana* is a single rosette and individuals may live several decades before their unique reproductive event. Plant senescence begins after flowering and most plants are dry by the time seeds are dispersed a year later (Sáyago 2016).

### Sampling design

We sampled *T. makoyana* and *T. intermedia* at three continuous and three fragmented tropical dry forest sites for 3 years (2008–10). To determine areas with the highest density of adult individuals, we conducted a preliminary survey of all trails within the Chamela-Cuixmala Biosphere Reserve (CCBR) and forest fragments within a 30 km radius from the biological station. We found both *Tillandsia* species co-occurring at three fragmented sites and at two continuous forest sites. Thus, we included two additional sites with a single species each within the continuous forest to have three replicate sites per species. The four study sites of continuous habitat were all within the large forested area that comprises the CCBR at least 200 m away from the forest edge (Fig. 1). Continuous forest sites shown near edges of the reserve are not actual forest edges, since the continuous forest extends beyond the official boundaries of the CCBR. The sites in closest proximity to each other were 2 km apart in a straight line, and the most distant ones were 13 km apart. The fragmented study sites were isolated remnants of old-growth forest trees including two to five adult phorophytes (400–700 m^2^), located within a matrix of human settlements, cultivated land, active cattle pastures and secondary growth; these sites were between 4 and 8 km away from the edges of the CCBR (Fig. 1).

Both *Tillandsia* species are generalist in terms of phorophyte use, occurring in at least 30 plant species distributed in 11–14 families; the most common phorophytes were *Apoplanesia paniculata* and *Cesalpinea eriostachys* for both species (Sáyago *et al.* 2013). At fragmented sites, reproductive individuals of the two *Tillandsia* species were selected from the few large trees that remained after forest clearance. These phorophyte species were a subsample of the phorophytes found within the continuous forest.

For both *Tillandsia* species, we selected reproductive individuals that could be accessed with ladders. Since *T. intermedia* is a clonal species, we chose individuals that were located on different branches of the tree that were clearly independent from other selected individuals. Reproductive individuals were identified by the presence of immature inflorescences.

### Breeding system

At two sites in the continuous forest during the flowering season of 2011, we determined the capacity for autonomous self-pollination in 98 plants of *T. intermedia* (Site C2 in Fig. 1) and 28 plants of *T. makoyana* (Sites C1 and C2 in Fig. 1). We contrasted the fruit set of bagged inflorescences with the fruit set of open-pollinated inflorescences on different plants (since rosettes produce a single inflorescence during the season). We calculated the autofertility index (AFI) as the fruit set of bagged flowers divided by the fruit set of open-pollinated flowers (modified from Lloyd and Schoen 1992).

### Pollinator visitation

We conducted pollinator observations during three consecutive years (2008–10). Overall, we observed 161 individuals of *T. intermedia* (59 in continuous forests and 102 in forest fragments) and 175 individuals of *T. makoyana* (101 in continuous forests and 74 in forest fragments). This work was conducted on 73 different days for a total of 483 h for *T. intermedia* and 525 h for *T. makoyana* with a Sony Digital Handycam DCR-PC 100 for 3-h periods distributed throughout the day from 0700 to 1900 h. We recorded the identity of the visitor, the duration of the visit, and whether or not it contacted stigmas and anthers. The two *Tillandsia* species had exerted reproductive organs; therefore, contact with the pollinator′s bill or body was always possible to determine from video recordings.

In 2008, we recorded visitation to flowers throughout day and night. However, no pollinator visits were recorded during night-time; therefore, the following years we only conducted observations during daytime. Pollinator visitation rates were estimated by dividing the total number of visits to an inflorescence by the number of hours observed; thus, we report number of visits per inflorescence per hour considering only daytime observations.

### Female reproductive success

To test for differences in reproductive success between continuous and fragmented habitats, we tagged one inflorescence on each of 703 individuals of *T. intermedia* (329 in continuous and 374 in fragmented) and 226 individuals of *T. makoyana* (158 in continuous and 68 in fragmented) over 3 years (2009–11). We quantified the total number of flowers and mature fruits produced per inflorescence. The fruit set of each plant was calculated as the total number of fruits divided by the total number of flowers produced per inflorescence each year.

Seed production was quantified from fruits collected in 2011 from 64 individuals of *T. intermedia* (35 in continuous and 29 in fragmented) and 65 individuals of *T. makoyana* (38 in continuous and 27 in fragmented). Seeds were counted on 1–3 fruits per individual, depending on fruit availability. Seeds were considered viable if they had a fully developed endosperm and coma (filamentous structure that allows dispersal and attachment to substrate). Smaller wrinkled seeds were classified as aborted seeds. The sum of viable and aborted seeds was considered the total number of ovules produced per ovary.

### Statistical analyses

Statistical analyses were conducted through generalized linear mixed models using PROC GLIMMIX in SAS (SAS Institute Inc. 2008). Since we were interested in understanding the temporal effect of habitat fragmentation on plant reproductive success, we included year (2008–10) and habitat condition (continuous vs. fragmented) as fixed factors. The interaction term between fixed factors was eliminated from the model because it was not statistically significant for either *Tillandsia* species. Site was considered a random factor nested within habitat condition. Response variables included: number of flowers, number of fruits, and pollinator visitation rates (following a Poisson distribution with a log link function), ovule number (following a normal distribution), and fruit and seed set (following a binomial distribution with a logit link function). Back-transformed means were obtained using the *ilink* function. *P*-values for multiple comparisons were Tukey-adjusted. For number of ovules and seed set (number of viable seeds/number of ovules) a simple analysis of variance was performed to assess differences between continuous and fragmented conditions, since these variables were only measured for a subset of individuals during 2011.

## Results

### Breeding system

In *T. intermedia*, the mean fruit set (±SEM) of open-pollinated inflorescences was 32 % (±12.1), and there was no fruit production in bagged inflorescences with pollinators excluded. In *T. makoyana*, the mean fruit set of open-pollinated inflorescences was 40 % (±10.6), while the fruit set of bagged inflorescences was 19 % (±4.8). The AFI, an estimate of the potential for autonomous self-pollination, was zero for *T. intermedia* and 0.47 for *T. makoyana*.

### Pollinator assemblages and visitation rates

Pollinator observations revealed that flowers of *T. intermedia* and *T. makoyana* are visited by hummingbirds and bees, but hummingbirds are the predominant pollinators under both landscape conditions. Hummingbirds generally visited flowers for 1–2 s and they contacted both stigmas and anthers on every visit, therefore, hummingbirds can be considered legitimate pollinators. Secondary pollinators included orchid bees (Euglossini) and stingless bees (Meliponini), the former being rare and present only in continuous forest sites. Stingless bees, the second most abundant group of floral visitors, contacted the reproductive structures 83 % of their visits and spent long periods of time on each flower (5.4 ± 2.8 minutes per visit). In all cases, bees moved to neighbouring flowers and plants on the same branch before moving out of sight.

Although pollinator functional groups did not differ between habitat conditions, the relative abundances of different visitors significantly changed with habitat fragmentation, particularly in the case of *T. intermedia* (Table 1). In this species, the frequency of visitation by stingless bees increased in forest fragments, as well as visitation by the broad-billed hummingbird *Cynanthus latirostris*, a species that was extremely rare at *Tillandsia* flowers in the continuous forest.

**Table 1.** Pollinator assemblages of *Tillandsia intermedia* and *T. makoyana* recorded during 2008–10 in continuous and fragmented tropical dry forest sites in the region of Chamela, Jalisco, Mexico. Values indicate percent number of visits by each animal taxon that contacted the reproductive organs of flowers. *N* is the total number of legitimate visits to inflorescences observed in each habitat condition. Analyses of differences in pollinator composition between continuous and fragmented habitats are shown for each *Tillandsia* species (***P < 0.0001).

	*T. intermedia*		*T. makoyana*	
Floral visitor	Continuous (*N* = 80)	Fragmented (*N* = 356)	Continuous (*N* = 1059)	Fragmented (*N* = 716)
*Amazilia rutila*	75	42	80	82
*Cynanthus latirostris*	0	21	1	2
*Heliomaster constantii*	0	2	0.5	4
*Euglossa* sp. (bee)	11	0	0.5	0
Meliponinae (bee)	14	35	18	12
Chi-square value	16.98***		0.02	

**Figure 3.**
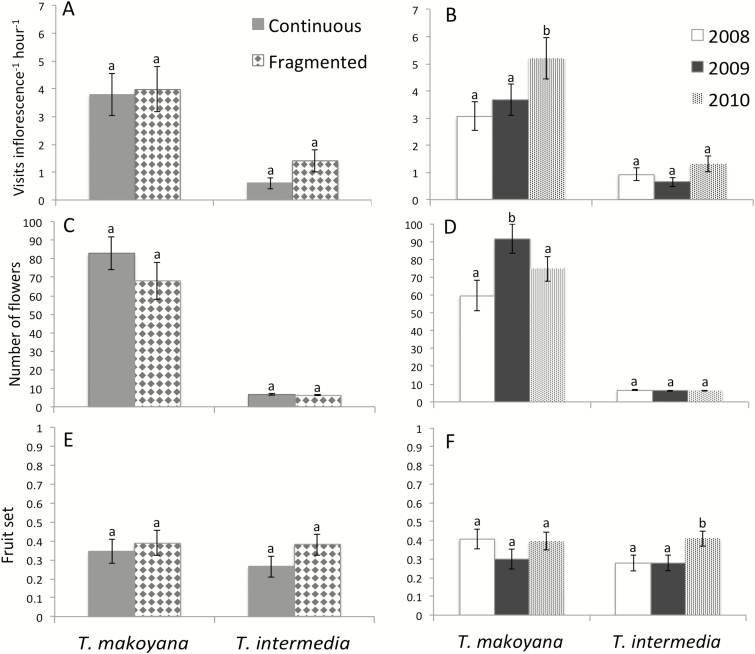
Pollinator visitation (visits per inflorescence per hour) (A and B), flower production per plant (B and C) and fruit production (E and F) of polycarpic *Tillandsia intermedia* and *T. makoyana* during 2008–10 at continuous and fragmented sites in the region of Chamela, Jalisco, Mexico.

Visitation rates (visits per inflorescence per hour) were similar between continuous and fragmented forests for both species (*T. intermedia F*_1, 4_ = 3.7, *P* = 0.13; *T. makoyana F*_1, 4_ = 0.03, *P* = 0.86; Fig. 3A), although there was a trend for higher visitation in forest fragments, particularly evident in *T. intermedia*. There was temporal variation for both species, with visitation being higher in 2010 than in previous years (*T. intermedia F*_2, 149_ = 6.64, *P* < 0.005; *T. makoyana F*_2, 167_ = 11.98, *P* < 0.0001; Fig. 3B).

### Female reproductive success

*Tillandsia makoyana* individuals produced 10 times as many flowers as *T. intermedia* plants in a single reproductive episode. Likewise, the number of fruits produced by *T. makoyana* individuals 10-folded the fruit production of *T. intermedia* individuals. In absolute terms, *T. makoyana* individuals produced on average 3800 seeds per ramet per reproductive episode, 14 times more seeds than *T. intermedia* individuals, which produced on average 260 seeds per ramet.

When comparing flower production per individual between habitat conditions, both species produced a similar number of flowers in continuous and fragmented forests (*T. intermedia F*_1, 4_ = 1.3, *P =* 0.32; *T. makoyana F*_1, 4_ = 0.3, *P* = 0.60; Fig. 3C). The temporal analysis indicated that flower production was similar across time for *T. intermedia* (*F*_2, 695_ = 2.0, *P* = 0.13; Fig. 3D), but differed between years for *T. makoyana*, with greater flower production in 2009 (*F*_2, 220_ = 8.1, *P* < 0.0005; Fig. 3D).

The number of fruits produced per inflorescence by *T. intermedia* was similar in continuous and fragmented forests (*F*_1, 4_ = 1.0, *P* = 0.37; Table 2), but it was higher in 2010 than in the previous 2 years (*F*_2, 695_ = 11.6, *P* < 0.0001). The proportion of flowers becoming fruits (fruit set) did not differ between continuous and fragmented forests for *T. intermedia* (*F*_1, 4_ = 2.2, *P* = 0.22; Fig. 3E), but fruit set was higher in 2010 than in previous years (*F*_2, 695_ = 17.7, *P* < 0.0001; Fig. 3F). The mean number of ovules did not differ between continuous and fragmented forests (*F*_1, 58_ = 1.22, *P =* 0.273; Table 2), nor did seed set (*F*_1, 58_ = 0.14, *P =* 0.714; Table 2).

**Table 2.** Mean number of ovules, proportion of viable seeds ± SE (%) produced during 2011 by *Tillandsia intermedia* and *T. makoyana* under continuous and fragmented conditions in the tropical dry forest of Chamela-Cuixmala, Mexico. Numbers in bold indicate significantly different means at *P* > 0.05.

	*T. intermedia*		*T. makoyana*	
	Continuous	Fragmented	Continuous	Fragmented
Ovules	173 ± 5.7	164 ± 6.1	171 ± 8.9	181 ± 9.9
Seed set	78 ± 2.8	79 ± 2.7	**86 ± 2.4**	**77 ± 4.1**

In *T. makoyana* individuals, the mean number of fruits produced per inflorescence was similar between habitat conditions (*F*_1, 4_ = 0.02, *P* = 0.893; Table 2), and years (*F*_2, 220_ = 0.3, *P* = 0.76). Fruit set did not differ between habitat conditions (*F*_1, 4_ = 0.25, *P* = 0.64; Fig. 3E), but differed between years (*F*_2, 220_ = 4.6, *P* = 0.01; Fig. 3F). Ovule number did not differ between conditions either (*F*_1, 60_ = 0.55, *P* = 0.460; Table 2); however, seed set was higher in continuous than in fragmented forest (*F*_1, 60_ = 4.3, *P* < 0.05; Table 2).

## Discussion

Habitat fragmentation generally has negative consequences on the reproductive success of plants; however, some species show positive or no responses to fragmentation (Aguilar *et al.* 2006, 2008). The few studies that include vascular epiphytes, all of them polycarpic species, have found contrasting results: some species are negatively affected by fragmentation (*Rhipsalis lumbricoides*, *Tillandsia ixioides*, Aizen and Feinsinger 1994; *Myrmecophila christinae*, *Oncidium ascendens*, Parra-Tabla *et al.* 2000,^2011^), while other species show temporal variation ranging from negative to positive effects across years (*Catasetum viridiflavum*, Murren 2002). Adding to this diversity of effects, this study showed that, despite differences in life history strategies, most reproductive variables in *T. intermedia* and *T. makoyana* did not differ between habitat conditions. The only variables that changed with fragmentation were the composition of pollinator assemblages in *T. intermedia*, and seed set in *T. makoyana*, which was lower in fragmented habitats. These results may be related to intrinsic plant traits such as breeding system and floral display, and to external factors such as the availability of resources or pollinators.

Plant reproductive systems are important to understand the impact of habitat fragmentation on plant fitness because they relate to the capacity of plants to set seed in the absence of conspecifics or pollinators (Aguilar *et al.* 2006, 2008). Epiphytism has been associated with self-compatible and autonomous breeding systems in various plant families (Bush and Beach 1995; Martén-Rodríguez *et al.* 2015); however, in the Bromeliaceae, both self-compatible and self-incompatible breeding systems have been documented (Matallana *et al.* 2010). Given that monocarpic species have a single opportunity to reproduce in a lifetime, self-pollination is expected to provide reproductive assurance in monocarpic epiphytes (Cole 1954). Consistent with this prediction, this study showed that only monocarpic *T. makoyana* is self-fertile, despite the fact that pollinator visitation rates were higher and floral displays larger than they were in *T. intermedia* (Fig. 3). In contrast, polycarpic *T. intermedia* showed no capacity for autonomous self-pollination; therefore, it is dependent on pollinators for reproduction. A greater effect of habitat fragmentation on plant female reproductive success is expected in self-incompatible and other pollinator-dependent plants, which more often have seed production limited by pollinators or pollen (Cunningham 2000; Aguilar *et al.* 2006). Surprisingly, female reproductive success in *T. intermedia* was similar between fragmented and continuous habitats, indicating that, at least during the three study years, pollinators were equally good at effecting fruit and seed set under both habitat conditions. Nevertheless, given that *T. intermedia* is self-incompatible and clonal, it is vulnerable to pollen limitation, particularly in habitats where pollinator populations are low or where they fluctuate in time. For instance, hurricanes have hit the study area twice in the past 8 years (Tapia-Palacios *et al.* 2018), causing changes in vegetation structure and declines in natural populations of bromeliads (R. Sáyago *et al.*, unpubl. data). Large storms could also cause temporal variation in pollinator visitation and increase pollen limitation in *T. intermedia*, but these questions require further study.

Results indicate that *T. intermedia* and *T. makoyana* are primarily pollinated by hummingbirds, as are many species in the family Bromeliaceae (Stiles 1975; Buzato *et al.* 2000; Kaehler *et al.* 2005). *Amazilia rutila*—the most common pollinator—has a bill length of 23 ± 1.3 mm (Arizmendi and Ornelas 1990); therefore, it is one of few species that can effectively access nectar from *T. intermedia* and *T. makoyana* flowers (mean ± SEM: 26 ± 0.6 and 29 ± 0.8 mm long, respectively), along with the cinnamon hummingbird *C. latirostris*, a slightly smaller species. Both hummingbird species are permanent residents of Chamela and they are both territorial, but *A. rutila* dominates most territorial interactions (Arizmendi and Ornelas 1990). *Cynanthus latirostris* is a particularly common species in disturbed environments and cities across dry and arid regions of Mexico (Arizmendi and Ornelas 1990; Arizmendi and Berlanga 2014). Consistently, *C. latirostris* had a relatively high visitation frequency to flowers of *T. intermedia* in forest fragments. The relative abundances of other floral visitors to *T. intermedia* also varied between habitat conditions; for instance, stingless bees increased their frequency in forest fragments, whereas Euglossine bees showed the opposite trend (Table 1). This change in relative visitation frequencies among pollinator species—only observed for *T. intermedia*—may be the result of different factors: (i) the populations of certain groups of floral visitors, such as Euglossine bees, are often negatively impacted by habitat loss or fragmentation (e.g. Storck-Tonon and Peres 2017); (ii) the movement of animal pollinators may be constrained by open spaces in fragmented habitats (Volpe *et al.* 2016); and (iii) pollinator behaviour may change along with vegetation structure and floral resources after fragmentation (Goverde *et al.* 2002; Hadley and Betts 2009). In this study, Euglossine bees did not visit *Tillandsia* flowers in forest fragments, and hummingbirds changed their frequencies according to habitat condition in *T. intermedia*. For instance, *C. latirostris* feeds from a variety of tree species whose flowers contain relatively high amounts of nectar in the continuous forest (Arizmendi and Ornelas 1990); however, in fragmented areas, this species uses lower rewarding *T. intermedia* flowers. A possible explanation for this result is that *T. intermedia* is clonal and occurs in high abundance on isolated trees in small forest fragments, flowering during the driest months of the year when other floral resources are scarce (Sáyago 2016). Tillandsias tend to occur in relatively high numbers on large- and medium-sized trees (Sáyago *et al.* 2013); therefore, the overall reward obtained by visiting multiple flowers on the same host tree possibly provides enough energy to hummingbird pollinators. This behaviour might cause limited pollen movement within forest fragments, high genetic structuring, and low gene flow between populations, a genetic pattern that has been observed in vertebrate-pollinated epiphytic bromeliads in fragmented habitats (González-Astorga *et al.* 2004; Paggi *et al.* 2015). Similarly, the foraging behaviour of stingless bees may promote self-pollination in forest fragments where these bees are more abundant; however, the genetic consequences associated with changes in pollinator composition and behaviour in fragmented landscapes are still unknown for Mexican dry forest tillandsias.

While the composition and frequency of pollinators of *T. intermedia* changed with forest fragmentation, overall pollinator visitation rates and fruit set were comparable among habitat conditions in both *Tillandsia* species. These results follow a general prediction previously stated in the literature that large-bodied pollinators (e.g. mainly vertebrates) are less affected by long distances between flower resources in remnant fragments than smaller pollinators (Ghazoul and Shaanker 2004). However, it has been demonstrated, particularly in rainforest environments, that some hummingbirds are exclusively forest species that are negatively affected by forest fragmentation, while others are capable of surviving and moving across anthropized landscapes or fragmented habitats (Stouffer and Bierregaard 1995; Hadley *et al.* 2018). The two hummingbird species that visited *Tillandsia* flowers in this study, *A. rutila* and *C. latirostris*, are altitudinal migrants and have broad geographic distributions across dry continuous and open habitats (Ornelas and Arizmendi-Arriaga 1995; Arizmendi and Berlanga 2014); therefore, their presence in the fragmented habitats of the Chamela region is another indicator that these hummingbird species can act as effective pollen vectors in disturbed environments.

Changes in the visitation frequencies and behaviours of different pollinator species between habitats could influence the quantity or quality of pollen received by flowers, which would likely affect plant reproductive success and progeny quality (Quesada *et al.* 2001; Cascante *et al.* 2002). In self-compatible species like *T. makoyana*, autonomous self-pollination might provide reproductive assurance, but this benefit could be countered by the negative effects of inbreeding on the progeny produced via self-pollination (Herlihy and Eckert 2004). The lower seed set of *T. makoyana* under fragmented habitat conditions might potentially be the result of increased inbreeding depression, since the main pollinator, *A. rutila*, is a highly territorial species (Arizmendi and Ornelas 1990), and bees tend to move between neighbouring flowers within the same host trees; these behaviours might promote geitonogamous crosses and perhaps crosses between related individuals (e.g. Feinsinger 1978). The few studies available on epiphytic bromeliads indicate low genetic variation and low outcrossing rates, in addition to limited gene flow and restricted neighbourhoods, particularly in self-pollinating species (Soltis *et al.* 1987; Gonzalez-Astorga *et al.* 2004; Cascante-Marín *et al.* 2014); however, for *T. makoyana* these ideas remain elusive. Another potential explanation for the reduced seed set of *T. makoyana* in forest fragments is that greater attack by herbivores and pathogens in forest fragments would lower resources for reproduction or directly affect the developing seeds. However, a recent meta-analysis shows that fragmentation generally does not cause an increase in herbivory and there was no evidence of herbivory or seed predation in the study species (Rossetti *et al.* 2017). In addition, herbivory levels are generally relatively low in epiphytes (Zotz 2016). An alternative explanation is that, since epiphytes are often nutrient-limited (Boelter *et al.* 2014), low resources for reproduction might reduce seed production in disturbed habitats because large isolated trees in fragmented habitats are more exposed to wind, desiccation and nutrient leaching (Flores-Palacios and García-Franco 2004).

 Despite observing similar female fruit set in continuous and fragmented habitat conditions, we should not infer that there are no fragmentation effects on demographic or genetic parameters of these *Tillandsia* species. It should be highlighted that the density of *T. makoyana* individuals is drastically reduced in fragmented and successional sites as a result of a reduction in the abundance of appropriate phorophytes in comparison to the continuous forests; for example, at continuous forest sites, the mean number of *T. makoyana* individuals in a sample of 100 m^2^ plots was 21 (SD = 26.1), while at successional/fragmented sites it was 0.2 (SD = 0.38) (Sáyago 2016). Likewise, disturbance has marked negative effects on the diversity and composition of the phorophyte community (Quesada *et al.* 2009; Munguía-Rosas and Montiel 2014), and in microclimatic conditions (Laurance 2004), which are critical for the survival of epiphytic species in dry forest habitats. Studies on epiphytic plants show contrasting results: some have found lower diversity but higher recruitment in secondary cloud forests (Cascante-Marín *et al.* 2006), or lower seedling establishment on isolated trees than in old-growth forests (Werner and Gradstein 2008), while other studies show that some rainforest epiphytes actually increase in abundance in human-modified environments (Einzmann and Zotz 2017). Nevertheless, for epiphytes that depend on trees with particular characteristics, such as dry forest *Tillandsia* species, the reduction in the abundance of appropriate phorophytes affects the recruitment of new plants and possibly the long-term viability of epiphyte populations (Sáyago *et al.* 2013). Under these conditions, the populations would not only experience greater environmental and demographic stochasticity, but they would also be vulnerable to reduction in genetic variation and increased inbreeding depression (Keller and Waller 2002).

In conclusion, this study demonstrates that pollinator communities significantly changed in species composition and relative abundances between habitat conditions in polycarpic *T. intermedia*, but they did not in monocarpic *T. makoyana.* In addition, habitat fragmentation was associated with a lower seed set in *T. makoyana* but not *T. intermedia.* However, fruit production was similar between habitat conditions for both *Tillandsia* species despite their different breeding systems and life history strategies. This result is possibly due to the fact that hummingbirds with wide geographic distributions and broad habitat use are the main pollinators of the study species. Future studies should address the consequences of habitat fragmentation and changes in pollinator communities and behaviour on genetic structuring, gene flow via pollen and seeds, levels of inbreeding and progeny quality.

## Sources of Funding

This work was supported by grants from Consejo Nacional de Ciencia y Tecnología, México (CONACYT Laboratorios Nacionales 293701 and Repositorio Institucional 271432), SAGARPA-CONACyT (291333), PAPIIT (IA208416, IA207618 to S.M.-R. and IV200418 to M.Q.).

## Contributions by the Authors

R.S. and M.Q. designed the study, performed fieldwork and data processing; R.A., M.L.-M. and L.A. contributed with fieldwork and data processing; S.M.-R. conducted statistical analyses and wrote the manuscript with contributions from all authors.

## Conflict of Interest

None declared.

## References

[CIT0001] Aguilar R, Ashworth L, Galetto L, Aizen MA 2006 Plant reproductive susceptibility to habitat fragmentation: review and synthesis through a meta-analysis. Ecology Letters 9:968–980. 16913941 10.1111/j.1461-0248.2006.00927.x

[CIT0002] Aguilar R, Quesada M, Ashworth L, Herrerias-Diego Y, Lobo J 2008 Genetic consequences of habitat fragmentation in plant populations: susceptible signals in plant traits and methodological approaches. Molecular Ecology 17:5177–5188. 19120995 10.1111/j.1365-294X.2008.03971.x

[CIT0003] Aguirre A, Guevara R, García M, López JC 2010 Fate of epiphytes on phorophytes with different architectural characteristics along the perturbation gradient of *Sabal mexicana* forests in Veracruz, Mexico. Journal of Vegetation Science 21:6–15.

[CIT0004] Aizen M, Feinsinger P 1994 Forest fragmentation, pollination and plant reproduction in a Chaco dry forest, Argentina. Ecology 75:330–351.

[CIT0005] Amasino R 2009 Floral induction and monocarpic versus polycarpic life histories. Genome Biology 10:228. 19591661 10.1186/gb-2009-10-7-228 PMC2728520

[CIT0006] Arizmendi MC, Berlanga H 2014 Colibríes de México y Norteamérica. México, D.F: CONABIO.

[CIT0007] Arizmendi C, Ornelas JF 1990 Hummingbirds and their floral resources in a tropical dry forest in Mexico. Biotropica 22:172–180.

[CIT0008] Bawa KS, Kang H, Grayum MH 2003 Relationships among time, frequency, and duration of flowering in tropical rain forest trees. American Journal of Botany 90:877–887. 21659182 10.3732/ajb.90.6.877

[CIT0009] Benzing DH 1990 Vascular epiphytes. Cambridge: Cambridge University Press.

[CIT0010] Benzing DH 2000 Bromeliaceae: profile of an adaptive radiation. Cambridge: Cambridge University Press.

[CIT0011] Boelter CR, Givnish TJ, Bermudes D 2014 A tangled web in tropical tree tops: effects of edaphic variation, neighborhood phorophyte composition and bark characteristics on epiphytes in a central Amazonian forest. Journal of Vegetation Science 25:1090–1099.

[CIT0012] Brys R, de Crop E, Hoffmann M, Jacquemyn H 2011 Importance of autonomous selfing is inversely related to population size and pollinator availability in a monocarpic plant. American Journal of Botany 98:1834–1840. 22003180 10.3732/ajb.1100154

[CIT0013] Bush SP, Beach JH 1995 Breeding systems of epiphytes in a tropical montane wet forest. Selbyana 16:155–158.

[CIT0014] Buzato S, Sazima M, Sazima I 2000 Hummingbird-pollinated floras at three Atlantic forest sites. Biotropica 32:824–841.

[CIT0015] Cascante A, Quesada M, Lobo JA, Fuchs EJ 2002 Effects of dry tropical forest fragmentation on the reproductive success and genetic structure of the tree *Samanea saman*. Conservation Biology 16:137–147. 10.1046/j.1523-1739.2002.00317.x 35701973

[CIT0016] Cascante-Marín A, Oostermeijer G, Wolf J, Fuchs EJ 2014 Genetic diversity and spatial genetic structure of an epiphytic bromeliad in Costa Rican montane secondary forest patches. Biotropica 46:425–432.

[CIT0017] Cascante-Marín A, von Meijenfeldt N, de Leeuw HM 2009 Dispersal limitation in epiphytic bromeliad communities in a Costa Rican fragmented montane landscape. Journal of Tropical Ecology 25:63–73.

[CIT2325] Cascante-Marín A, Wolf JHD, Oostermeijer JGB, dens Nijs JCM, Sanahuja I, Duran-Apuy A 2006 Epiphytic bromeliad communities in secondary and mature forest in a tropical premontane area. Basic and Applied Ecology 7:520–532.

[CIT0018] Cole LC 1954 The population consequences of life history phenomena. The Quarterly Review of Biology 29:103–137. 13177850 10.1086/400074

[CIT0019] Cunningham SA 2000 Depressed pollination in habitat fragments causes low fruit set. Proceedings of the Royal Society of London B 267:1149–1152. 10.1098/rspb.2000.1121 PMC1690653 10885521

[CIT0020] Einzmann HJR, Zotz G 2017 No signs of saturation: long-term dynamics of vascular epiphyte communities in a human-modified landscape. Biodiversity Conservation 26:1393.

[CIT0021] Fahrig L 2004 Effects of habitat fragmentation on biodiversity. Annual Review of Ecology, Evolution and Systematics 34:487–515.

[CIT0022] Feinsinger P 1978 Ecological interactions between plants and hummingbirds in a successional tropical community. Ecological Monographs 48:269–287.

[CIT0023] Flores-Palacios A, García-Franco JG 2004 Effect of isolation on the structure and nutrient content of oak epiphyte communities. Plant Ecology 173:259–269.

[CIT0024] Fuchs EJ, Lobo JA, Quesada M 2003 Effects of forest fragmentation and flowering phenology on the reproductive success and mating patterns of the tropical dry forest tree *Pachira quinata*. Conservation Biology 17:149–157.

[CIT0025] García-Oliva F, Camou A, Mass JM 2002 El clima de la región central de la costa del Pacífico mexicano. In: Noguera F, Vega J, García A, Quesada M, eds. Historia natural de Chamela. México: Universidad Nacional Autónoma de México, 3–10.

[CIT0026] Gentry A 1995 Diversity and floristic composition of neotropical dry forests. In: Bullock S, Mooney H, Medina E, eds. Seasonally dry tropical forests. Cambridge: Cambridge University Press, 146–194.

[CIT0027] Ghazoul J, Shaanker RU 2004 Sex in space: pollination among spatially isolated plants. Biotropica 36:128–130.

[CIT0028] González-Astorga J, Cruz-Angón A, Flores-Palacios A, Vovides AP 2004 Diversity and genetic structure of the Mexican endemic epiphyte *Tillandsia achyrostachys* E. Morr. ex Baker var. *achyrostachys* (Bromeliaceae). Annals of Botany 94:545–551. 15319228 10.1093/aob/mch171 PMC4242225

[CIT0029] Goverde M, Schweizer K, Baur B, Erhardt A 2002 Small-scale habitat fragmentation effects on pollinator behaviour: experimental evidence from the bumblebee *Bombus veteranus* on calcareous grasslands. Biological Conservation 104:293–299.

[CIT0030] Hadley AS, Betts MG 2009 Tropical deforestation alters hummingbird movement patterns. Biology Letters 5:207–210. 19158031 10.1098/rsbl.2008.0691 PMC2665823

[CIT0031] Hadley AS, Frey SJ, Robinson WD, Betts MG 2018 Forest fragmentation and loss reduce richness, availability, and specialization in tropical hummingbird communities. Biotropica 50:74–83.

[CIT0032] Herlihy CR, Eckert CG 2004 Experimental dissection of inbreeding and its adaptive significance in a flowering plant, *Aquilegia canadensis* (Ranunculaceae). Evolution 58:2693–2703. 15696748 10.1111/j.0014-3820.2004.tb01622.x

[CIT0033] Hofstede RGM, Wolf JHD, Benzing DH 1993 Epiphytic biomass and nutrient status of a Colombian upper montane rain forest. Selbyana 14:37–45.

[CIT0034] Honnay O, Jacquemyn H, Bossuyt B, Hermy M 2005 Forest fragmentation effects on patch occupancy and population viability of herbaceous plant species. The New Phytologist 166:723–736. 15869637 10.1111/j.1469-8137.2005.01352.x

[CIT0035] Kaehler M, Varassin IG, Goldenberg R 2005 Polinizacão em uma comunidade de bromélias em floresta atlantica alto-montana no Estado do Paraná, Brasil. Revista Brasileira de Botanica 28:219–228.

[CIT0036] Keller LF, Waller DM 2002 Inbreeding effects in wild populations. Trends in Ecology and Evolution 17:230–241.

[CIT0037] Laurance WF 2004 Forest-climate interactions in fragmented tropical landscapes. Philosophical Transactions of the Royal Society of London. Series B, Biological Sciences 359:345–352. 15212089 10.1098/rstb.2003.1430 PMC1693331

[CIT0038] Lloyd DG, Schoen DJ 1992 Self- and cross-fertilization in plants. I. Functional dimensions. International Journal of Plant Sciences 153:358–369.

[CIT0039] Lott EJ, Atkinson TH 2006 Mexican and Central American seasonally dry tropical forests: Chamela-Cuixmala, Jalisco, as focal point for comparison. In: Pennington RT, Lewis GP, Ratter JA, eds. Neotropical savannas and seasonally dry forests: plant diversity, biogeography and conservation. Boca Raton, FL: CRC Press, 307–334.

[CIT0040] Magrach A, Larrinaga AR, Santamaría L 2012 Internal habitat quality determines the effects of fragmentation on austral forest climbing and epiphytic angiosperms. PLoS One 7:e48743. 23119096 10.1371/journal.pone.0048743 PMC3485344

[CIT0041] Martén-Rodríguez S, Quesada M, Almarales-Castro A, Lopezaraiza‐Mikel M, Fenster CB 2015 A comparison of reproductive strategies between island and mainland Caribbean Gesneriaceae. Journal of Ecology 5:1190–1204.

[CIT0042] Martin PH, Sherman RE, Fahey TJ 2004 Forty years of tropical forest recovery from agriculture: structure and floristics of secondary and old-growth riparian forests in the Dominican Republic. Biotropica 36:297–317.

[CIT0043] Matallana G, Godinho MAS, Guilherme FAG, Belisario M, Coser TS, Wendt T 2010 Breeding systems of Bromeliaceae species: evolution of selfing in the context of sympatric occurrence. Plant Systematics and Evolution 289:57–65.

[CIT0044] Mix C, Pico FX, Van Groenendael JM, Ouborg NJ 2006 Inbreeding and soil conditions affect dispersal and components of performance of two plant species in fragmented landscapes. Basic Applied Ecology 7:59–69.

[CIT0045] Munguía-Rosas MA, Montiel S 2014 Patch size and isolation predict plant species density in a naturally fragmented forest. PLoS One 9:e111742. 25347818 10.1371/journal.pone.0111742 PMC4210240

[CIT0046] Murren C 2002 Effects of habitat fragmentation on pollination: pollinators, pollinia viability and reproductive success. Journal of Ecology 90:100–107.

[CIT0047] Ornelas JF, Arizmendi-Arriaga MC. 1995 Altitudinal migration: implications for conservation of avian neotropical migrants in western Mexico. In: Wilson MH, Sader SA, eds. Conservation of neotropical migratory birds in Mexico. Orono, ME: Maine Agricultural and Forest Experiment Station, 98–112.

[CIT0048] Paggi GM, Palma‐Silva C, Bodanese‐Zanettini MH, Lexer C, Bered F 2015 Limited pollen flow and high selfing rates toward geographic range limit in an Atlantic forest bromeliad. Flora, Morphology, Distribution, Functional Ecology of Plants 211:1–10.

[CIT0049] Parra-Tabla V, Vargas CF, Magaña-Rueda S, Navarro J 2000 Female and male pollination success of *Oncidium ascendens* Lindey (Orchidaceae) in two contrasting habitat patches: forest vs. agricultural field. Biological Conservation 94:335–340.

[CIT0050] Parra-Tabla V, Vargas CF, Naval C, Calvo LM, Ollerton J 2011 Population status and reproductive success of an endangered epiphytic orchid in a fragmented landscape. Biotropica 43:640–647.

[CIT0051] Poorter L, Zuidema PA, Peña-Clara M, Boot RGA 2005 A monocarpic tree species in a polycarpic world: how can *Tachigali vasquezii* maintain itself so successfully in a tropical rain forest community?Journal of Ecology 93:268–278.

[CIT0052] Quesada M, Aguilar R, Rosas F, Ashworth L, Rosas-Guerrero V, Sáyago R, Lobo JA, Herrerías-Diego Y, Sánchez-Montoya G 2011 Human impacts on pollination, reproduction and breeding systems in tropical forest plants. In: Dirzo R, Mooney H, Ceballos G, eds. Seasonally dry tropical forests. Washington, DC: Island Press, 173–194.

[CIT0053] Quesada M, Alvarez-Añorve M, Avila-Cabadilla L, Castillo A, Lopezaraiza-Mikel M, Martén-Rodríguez S, Rosas-Guerrero V, Sáyago R, Sánchez-Montoya G, Contreras-Sánchez JM, Balvino-Olvera F, Olvera-García SR, Lopez-Valencia S, Valdespino-Vázquez N 2014 Tropical dry forest ecological succession in México: synthesis of a long-term study. In: Sánchez-Azofeifa A, Powers J, Fernandes GW, Quesada M, eds. Tropical dry forests in the Americas: ecology, conservation and management. New York: CRC Press, 17–34.

[CIT0054] Quesada M, Fuchs EJ, Lobo JA 2001 Pollen load size, reproductive success, and progeny kinship of naturally pollinated flowers of the tropical dry forest tree *Pachira quinata* (Bombacaceae). American Journal of Botany 88:2113–2118. 21669642

[CIT0055] Quesada M, Sanchez-Azofeifa A, Alvarez-Añorve M, Stoner K, Avila-Cabadilla L, Calvo-Alvarado J, Castillo A, Espírito-Santo MM, Fagundes M, Fernandes GW, Gamon J, Lopezaraiza-Mikel M, Lawrence D, Cerdeira-Morellato LP, Powers JS, de S Neves F, Rosas-Guerrero V, Sáyago R, Sanchez-Montoya G 2009 Succession and management of tropical dry forests in the Americas: review and new perspectives. Forest Ecology and Management 6:1014–1024.

[CIT0056] Quesada M, Stoner KE 2004 Threats to the conservation of the tropical dry forest in Costa Rica. In: Frankie GW, Mata A, Vinson SB, eds. Biodiversity conservation in Costa Rica: learning the lessons in a seasonal dry forest. Berkeley, CA: University of California Press, 266–280.

[CIT0058] Rossetti MR, Tscharntke T, Aguilar R, Batáry P 2017 Responses of insect herbivores and herbivory to habitat fragmentation: a hierarchical meta-analysis. Ecology Letters 20:264–272. 28111900 10.1111/ele.12723

[CIT0059] Sanchez-Azofeifa A, Quesada M, Cuevas-Reyes P, Castillo A, Sánchez G 2008 Land cover and conservation in the area of influence of the Chamela-Cuixmala. Forest Ecology and Management 258:907–912.

[CIT0060] SAS Institute Inc 2008 SAS/STAT® 9.2 User’s Guide. Cary, NC: SAS Institute Inc.

[CIT0061] Sáyago R 2016 Ecología y conservación de las interacciones entre epífitas del género Tillandsia (Bromeliaceae), sus árboles hospederos y sus polinizadores en un bosque tropical seco. PhD Thesis, Universidad Nacional Autónoma de México, México.

[CIT0062] Sáyago R, Lopezaraiza-Mikel M, Quesada M, Álvarez-Añorve MY, Cascante-Marín A, Bastida JM 2013 Evaluating factors that predict the structure of a commensalistic epiphyte–phorophyte network. Proceedings of the Royal Society of London B 280:20122821. 10.1098/rspb.2012.2821 PMC3574374 23407832

[CIT0063] Soltis DE, Gilmartin AJ, Rieseberg L, Gardner S 1987 Genetic variation in the epiphytes *Tillandsia ionantha* and *T. recurvata* (Bromeliaceae). American Journal of Botany 74:531–537.

[CIT0064] Stiles FG 1975 Ecology, flowering phenology, and hummingbird pollination of some Costa Rican *Heliconia* species. Science 198:1177–1178.

[CIT0065] Storck-Tonon D, Peres CA 2017 Forest patch isolation drives local extinctions of Amazonian orchid bees in a 26 years old archipelago. Biological Conservation 214:270–277.

[CIT0066] Stouffer PC, Bierregaard RO 1995 Effects of forest fragmentation on understory hummingbirds in Amazonian Brazil. Conservation Biology 9:1085–1094. 10.1046/j.1523-1739.1995.9051072.x-i1 34261245

[CIT0067] Tapia-Palacios M, García-Suárez O, Sotomayor-Bonilla J, Silva-Magaña M, Pérez-Ortíz G, Espinosa-García A, Ortega-Huerta M, Díaz-Ávalos C, Suzán G, Mazari-Hiriart M 2018 Abiotic and biotic changes at the basin scale in a tropical dry forest landscape after Hurricanes Jova and Patricia in Jalisco, Mexico. Forest Ecology and Management 426:18–26.

[CIT0068] Trejo I, Dirzo R 2000 Deforestation of seasonally dry tropical forest: a national and local analysis in México. Biological Conservation 94:133–142.

[CIT0069] Turner IM, Tan HTW, Wee YC, Ibrahim AB 1994 A study of plant species extinction in Singapore: lessons for the conservation of tropical biodiversity. Conservation Biology 8:705–712.

[CIT0070] Volpe NL, Robinson WD, Frey SJ, Hadley AS, Betts MG 2016 Tropical forest fragmentation limits movements, but not occurrence of a generalist pollinator species. PLoS One 11:e0167513. 27941984 10.1371/journal.pone.0167513 PMC5152895

[CIT0071] Werner FA, Gradstein SR 2008 Seedling establishment of vascular epiphytes on isolated and enclosed forest trees in an Andean landscape, Ecuador. Biodiversity and Conservation 17:3195.

[CIT0072] Werner FA, Gradstein SR 2009 Diversity of dry forest epiphytes along a gradient of human disturbance in the tropical Andes. Journal of Vegetation Science 20:59–68.

[CIT0073] Young TP, Augspurger CK 1991 Ecology and evolution of long-lived semelparous plants. Trends in Ecology & Evolution 6:285–289. 21232483 10.1016/0169-5347(91)90006-J

[CIT0074] Zotz G 2016 Plants on plants-the biology of vascular epiphytes. Heidelberg, Berlin: Springer.

[CIT0075] Zotz G, Bader MY 2009 Epiphytic plants in a changing world: global change effects on vascular and non-vascular epiphytes. In: Lüttge U, Cánovas FM, Matyssek R, eds. Progress in Botany. Berlin: Springer Verlag, 147–170.

